# Age, gender, insulin and blood glucose control status alter the risk of ischemic heart disease and stroke among elderly diabetic patients

**DOI:** 10.1186/1475-2840-10-86

**Published:** 2011-10-06

**Authors:** Toshio Hayashi, Seinosuke Kawashima, Hideki Nomura, Hideki Itoh, Hiroshi Watanabe, Takashi Ohrui, Koutaro Yokote, Hirohito Sone, Yoshiyuki Hattori, Masao Yoshizumi, Koichiro Ina, Kiyoshi Kubota

**Affiliations:** 1 Department of Geriatrics, Nagoya University Graduate School of Medicine, Nagoya, Japan; 2 Division of Internal Medicine, Nakatsu Saiseikai Hospital, Osaka, Japan; 3 Department of Geriatrics, Nagoya Ajima Clinics, Nagoya, Japan; 4 Tokyo Metropolitan Geriatric Hospital, Tokyo, Japan; 5 Department of Clinical Pharmacology and Therapeutics, Hamamatsu University School of Medicine, Hamamatsu, Japan; 6 Department of Geriatric Medicine, Tohoku University School of Medicine, Sendai, Japan; 7 Division of Diabetes, Metabolism and Endocrinology, Department of Internal Medicine, Chiba University Graduate School of Medicine, Chiba, Japan; 8 Department of Endocrinology and Metabolism, University of Tsukuba Mito Medical Center, Mito, Japan; 9 Department of Endocrinology and Metabolism, Dokkyo University School of Medicine, Mibu, Japan; 10 Department of Cardiovascular Physiology and Medicine, Graduate School of Biomedical Science, Hiroshima University, Hiroshima, Japan; 11 Department of Phamacoepdiemiology, Graduate School of Medicine and Faculty of Medicine, the University of Tokyo, Japan

**Keywords:** Elderly, Diabetes mellitus, Insulin, Cerebral ischemia, Ischemic heart disease

## Abstract

**Background:**

We analyzed the effects of insulin therapy, age and gender on the risk of ischemic heart disease (IHD) and cerebrovascular accident (CVA) according to glycemic control.

**Methods and Results:**

We performed a prospective cohort study (Japan Cholesterol and Diabetes Mellitus Study) of type 2 diabetes patients (n = 4014) for 2 years. The primary endpoint was the onset of fatal/non-fatal IHD and/or CVA, which occurred at rates of 7.9 and 7.2 per 1000 person-years, respectively. We divided diabetic patients into four groups based on age (≤ 70 and > 70) and hemoglobin A1C levels (≤ 7.0 and > 7.0%). Multiple regression analysis revealed that IHD was associated with high systolic blood pressure and low HDL-C in patients under 70 years of age with fair glycemic control and was associated with low diastolic blood pressure in the older/fair group. Interestingly, insulin use was associated with IHD in the older/poor group (OR = 2.27, 95% CI = 1.11-5.89; p = 0.026) and was associated with CVA in the older/fair group (OR = 2.09, 95% CI = 1.06-4.25; p = 0.028). CVA was associated with lower HDL-C and longer duration of diabetes in younger/poor glycemic control group. Results by stepwise analysis were similar. Next, patients were divided into four groups based on gender and diabetic control(hemoglobinA1C < or > 7.0%). Multiple regression analysis revealed that IHD was associated with high systolic blood pressure in male/fair glycemic control group, age in male/poor control group, and short duration of diabetic history in females in both glycemic control groups. Interestingly, insulin use was associated with IHD in the male/poor group(OR = 4.11, 95% CI = 1.22-8.12; p = 0.018) and with CVA in the female/poor group(OR = 3.26, 95% CI = 1.12-6.24; p = 0.02). CVA was associated with short duration of diabetes in both female groups.

**Conclusions:**

IHD and CVA risks are affected by specific factors in diabetics, such as treatment, gender and age. Specifically, insulin use has a potential role in preventing IHD but may also be a risk factor for CVA among the diabetic elderly, thus revealing a need to develop improved treatment strategies for diabetes in elderly patients. The Japan Cholesterol and Diabetes Mellitus Study was formulated to evaluate them(Umin Clinical Trials Registry, clinical trial reg. no. UMIN00000516; http://www.umin.ac.jp/ctr/index.htm).

## Background

Elderly patients with type 2 diabetes mellitus (T2DM) have much higher risks of ischemic heart disease (IHD) and cerebrovascular accident (CVA) compared to younger diabetic patients. Because of these risks, diabetes may shorten an individual's life span by approximately 10 years [1]. A considerable number of studies have assessed IHD and CVA risk factors in culturally diverse groups of diabetic patients less than 70 years of age. With regard to glycemic control, a recent meta-analysis of several large clinical studies revealed that intensive and strict glycemic control was more effective than standard control for preventing IHD [2]. This review analyzed five trials, including the United Kingdom Prospective Diabetes Study (UKPDS), Action to Control Cardiovascular Risk in Diabetes (ACCORD), and Action in Diabetes and Vascular Disease: Preterax and Diamicron Modified Release Controlled Evaluation (ADVANCE). The meta-analysis study concluded that intensive glucose control [decreasing hemoglobin A1C (HbA1C) levels by 0.9%] was superior to standard control for preventing IHD. However, intensive glucose control did not seem to have any effect on stroke rates or overall survival [2-5]. Furthermore, most studies focused exclusively on patients under the age of 70 and did not examine elderly diabetic patients. Additionally, the authors did not evaluate whether specific diabetes treatments, such as insulin, had any effect on the risk of IHD and CVA.

The Japanese population has lower rates of IHD and higher rates of CVA than the U.S. and European populations [6]. However, the rate of IHD is much higher among Japanese diagnosed with diabetes [6,7]. Although it has been shown that elderly diabetic individuals have a higher risk of IHD than younger, non-diabetic patients, there is insufficient evidence regarding the associations between age, diabetic control, CVA, and IHD [8]. The present study, the Japan-CDM (Japan Cholesterol and Diabetes Mellitus Investigation), was a nationwide observational cohort study that enrolled 4,014 Japanese individuals with diabetes [7]. We recently reported the possibility of a change in the relationship between atherosclerotic risk factors and IHD or CVA based on age [9]. In other words, we identified a significant relationship between lower HDL or higher LDL cholesterol levels and the occurrence of IHD in subjects older than 65 years old. Lower HDL cholesterol was also significantly related to CVD in subjects over 65 years of age and especially in those older than 75. Lower HDL cholesterol is an important risk factor for IHD and CVD, especially in diabetic elderly individuals. Based on these data, the goal of this study is to evaluate the relationships between age, diabetic control, CVA and IHD in Japanese T2DM patients.

## Methods

### Patients

We recruited diabetic individuals examined at 40 institutions throughout Japan between September 2004 and March 2005. Patients who had experienced previous myocardial infarctions or cerebrovascular accidents requiring hospitalization were excluded from the study. Other exclusion criteria included the following: a history of or complications related to serious heart disease (such as acute heart failure); serious hepatic or renal disease, such as non-compensated liver cirrhosis and chronic renal failure requiring hemodialysis; malignancy; intention to undergo surgery; any illness with a poor prognosis; and the recruiting physician's judgment that a patient was inappropriate for inclusion in the study.

### Study design

**Figure 1:**
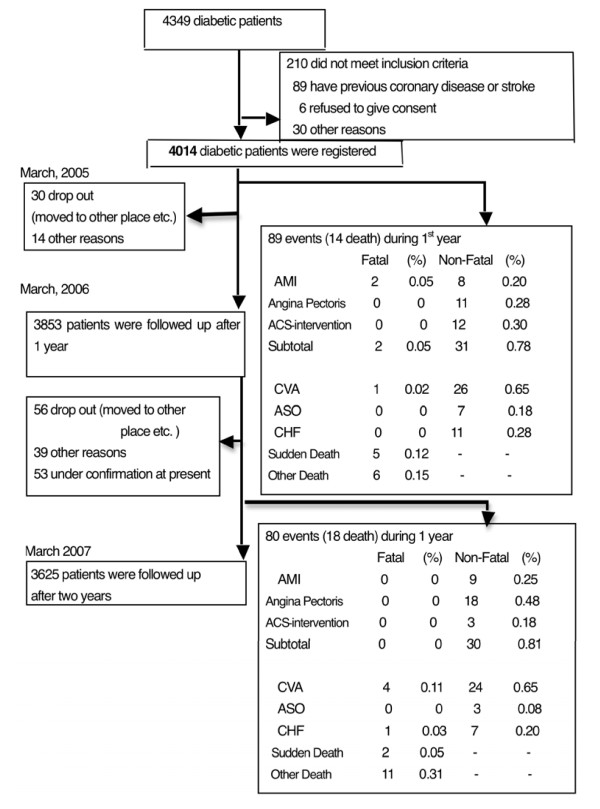
**Trial design and cardiovascular events that occurred after the first and second years among the diabetic individuals included in the study**. AMI: acute myocardial infarction, ACS: acute coronary syndrome, CVA: cerebrovascular accident (stroke), ASO: arteriosclerosis obliterans, CHF: congestive heart failure.

This multicenter prospective longitudinal cohort study included 4,014 diabetic individuals examined on a consecutive outpatient basis (1,936 women and 2,078 men; mean age = 67.4 ± 9.5 years, range = 35-83 years, median = 70.4 years). The one-year and two-year follow-up rates were 98.2% and 92.3%, respectively (Figure 1). Primary endpoints were the onset of IHD (comprising fatal or nonfatal myocardial infarction; development of unstable angina; or the need for coronary revascularization procedures, either coronary artery bypass grafting or percutaneous coronary intervention because of angina or an acute coronary syndrome) or CVA (stroke with neurological deficit, except transient ischemic attack). Secondary endpoints were sudden cardiac death due to causes other than myocardial infarction, transient ischemic attack, subarachnoid hemorrhage and all-cause mortality. We collected blood samples from the patients to evaluate their plasma lipid levels, blood glucose levels and HbA1C levels during the same month each year. Informed consent was obtained from all subjects participating in the study. The study was approved by the Institutional Review Boards of the participating hospitals and the relevant safety monitoring boards [10]. The guidelines of the Japan Atherosclerosis Society, which state that LDL values should be less than 120 mg/dL and HDL values should be higher than 40 mg/dL in diabetic individuals, and the diagnostic criteria for T2DM of the American Diabetes Association were used [11,12]. All reported events were confirmed by the organizing committee.

### Group allocation by age and glycemic control

We investigated the relationships between age, diabetic control, IHD, and CVA. Because the median age and hemoglobin A1C values of the patients in this study were 70.4 years and 7.0%, respectively, we divided the patients into four groups based on age (≤ 70 and > 70) and hemoglobin A1C levels (≤ 7.0 and > 7.0%). The four groups were as follows: 1) NF: under 70 years of age with fair glycemic control (n = 870); 2) NP: under 70 years of age with poor glycemic control (n = 1,072); 3) OF: over 70 years of age with fair glycemic control (n = 923); and 4) OP: over 70 years of age with poor glycemic control (n = 1,149).

### Group allocation by gender and glycemic control

We investigated the relationships between gender, diabetic control, IHD, and CVA. We divided the patients into four groups based on gender and hemoglobin A1C levels (≤ 7.0 and > 7.0%). The four groups were as follows: 1) MF: males with fair glycemic control (n = 1063); 2) MP: males with poor glycemic control (n = 950); 3) FF: females with fair glycemic control (n = 859); and 4) FP: females with poor glycemic control (n = 1,142).

### Statistics

Results are presented as means ± SD (standard deviation) of the data analyzed. All statistical analyses were performed using JMP (version 7, SAS Institute Inc., Cary, NC). The incidence of IHD and CVA were analyzed according to age, which was stratified as 70 years or younger vs. older than 70 years. We used the chi-square test and Fisher's exact test for categorical comparisons of the data. Differences in the means of continuous measurements were tested using the Mann-Whitney U test. We selected possible significant predictors by conducting a univariate logistic regression analysis (P < 0.10) and performed multiple logistic regression analysis to identify important risk factors for IHD and CVA. Additionally, stepwise regression analysis was used to confirm the relationships between IHD, CVA, and clinical variables. P-values less than 0.05 were considered to be statistically significant.

## Results

A total of 152 cardiovascular events (IHD and CVA) and 17 deaths due to other etiologies occurred during the 2-year study period. IHD and CVA occurred at rates of 7.9 and 7.2 per 1000 person-years, respectively. Table 1 shows the baseline characteristics of the four patient groups. In the NF group, fasting blood glucose levels, diastolic blood pressure readings and the female/male ratio were higher among patients using insulin than among patients not using insulin. Conversely, LDL-C was lower and HDL-C tended to be higher among patients using insulin than those not using insulin in the NF group. In the NP group, triglyceride and LDL-C levels were lower and HDL-C levels were higher among patients using insulin. In both the NF and NP groups, the history of diabetes was longer for patients using insulin. In both the OF and OP groups, the duration of diabetes was not different for insulin users compared to non-users. In the OP group, more females than males used insulin. We adjusted the data for these baseline differences in the following analyses.

**Table 1:** Base line profile of patients

	HbA1C = < 7.0 (Gp.MF)			HbA1C 7.0 < (Gp.MP)		
	
**= < 70y.o**.	Insulin (-)	Insulin (+)	P	Insulin (-)	Insulin (+)	P
				
	Mean(SD)	Mean(SD)		Mean(SD)	Mean(SD)	
Duration of Diabetes (yrs)	6.67 (7.50)	12.18 (9.31)	0.012*	9.53(8.05)	12.48 (8.15)	0.011*

FBS (mg/dl)	145.2 (44.4)	158.2 (55)	0.022*	183.7(63.4)	173.7(71)	0.134

SBP (mmHg)	134.4 (15.5)	133.4 (17.6)	0.409	133.4(17.7)	132.7 (18.3)	0.893

DBP (mmHg)	77 (12.3)	73.2 (14.8)	0.011*	76 (10.9)	73.4(11)	0.04*

TG (mg/dl)	148.3(119.9)	135.5 (65.3)	0.844	154.1(111.6)	141.9 (184)	0.03*

HDL-C(mg/dl)	55(14.2)	57.2 (15.1)	0.062	52.8(13.4)	59.7(21.5)	0.001**

LDL-C(mg/dl)	116.3(31.9)	110.5 (27.9)	0.034*	123.4(35.9)	120.2(33.3)	0.52

	%	%	P	%	%	P

**Gender (Male/total)**	61.8	49.6	0.011*	53.3	47.5	0.116

**Anti-hyperlipidemic drugs**	53.9	49.3	0.131	52.7	58.6	0.477

**statin**	41.4	39.0	0.122	48.5	45.7	0.444

**Oral diabetic drugs**	44.4	10	< 0.001**	82.0	6.7	< 0.001**

**sulfonylurea**	43.0	9.0	< 0.001**	79.4	8.6	< 0.001**

**Anti-hypertensive drugs**	66.1	47.9	0.042*	54.8	51.0	0.921

**70 +y.o**.	**HbA1C = < 7.0 (Gp.OF)**			**HbA1C 7.0 < (Gp.OP)**		
	
	**Insulin (-)**	**Insulin (+)**	**P**	**Insulin (-)**	**Insulin (+)**	**P**
				
	**Mean(SD)**	**Mean(SD)**		**Mean(SD)**	**Mean(SD)**	

Duration of Diabetes (yrs)	10.83 (8.75)	12.2 (9.67)	0.12	10.67 (9.17)	11.0 (9.50)	0.43

FBS (mg/dl)	146.2 (47.1)	159.1 (59)	0.052	174.5 (70.5)	181.7 (75.5)	0.224

SBP (mmHg)	136.2 (16.4)	135.7 (17)	0.896	136.4 (15.9)	136.2 (17)	0.822

DBP (mmHg)	71.7 (11.2)	70.4 (11.6)	0.256	73.1 (11.3)	73.1 (11.1)	0.832

TG (mg/dl)	127.5 (73.4)	124.3 (75.3)	0.168	130.8 (75)	124.6 (69.3)	0.53

HDL-C (mg/dl)	55.1 (16)	55 (17.3)	0.953	54.7 (16.1)	54.7 (16.5)	0.888

LDL-C (mg/dl)	114.1 (29)	112.3 (30.3)	0.704	118.2 (30.7)	117.8 (33.6)	0.541

	%	%	P	%	%	P

**Gender (Male/total)**	54	45.9	0.061	53.2	37.9	0.035*

**Anti-hyperlipidemic drugs**	50.2	46.0	0.140	46.7	50	0.307

**statin**	43.9	40.2	0.003**	43.3	42.5	0.167

**Oral diabetic drugs**	62.6	18.5	< 0.001**	84.6	19.1	< 0.001**

**sulfonylurea**	60.1	12.6	< 0.001**	79.4	8.0	< 0.001**

**Anti-hypertensive drugs**	68.2	59.9	0.904	60.9	54.6	0.370

Characteristics of the patients in each of the four groups, including insulin use. These groups were stratified by glycemic control and age.

*p < 0.05, **p < 0.01.

### Multiple regression analysis examining the relationship between the risk of IHD and clinical variables for each group divided by age and glucose control

Table 2 shows the relationship between IHD and each clinical measurement, such as LDL-C level and insulin treatment for each group divided by age and glucose control levels (HbA1C). We performed multiple regression analysis for variables that showed a possible relationship with IHD risk by univariate analysis (P < 0.1).

**Table 2:** The relationship between IHD, CVA and clinical variables

**IHD = < 70 y.o**.	Mean(SD)	Hgb A1C = < 7.0 (Gp.MF)		7.0 < HgbA1C (Gp.MP)	
**Number of events**		**17**		**15**	

		**Univariate**	**Multivariate**	**Univariate**	**Multivariate**

Male/Female	0.96	0.75 (0.40-1.37)		1.03 (0.62-1.74)	

Age (y.o.)	60.9(7.9)y.o.	1.06 (0.98-1.20)		1.03 (0.96-1.13)	

Duration of diabetes (years)	9.15(8.22)	1.04^+ ^(0.99-1.09)		1.03 (0.96-1.08)	

**Hemoglobin A1C(%)**	7.38(1.31)	1.16 (0.40-4.37)		1.01 (0.60-1.50)	

Triglyceride (mg/dl)	146.9(129.5)	1.01 (0.99 - 1.03)		1.01 (0.94-1.04)	

**LDL-Chol (mg/dl)**	119.2(34.1)	1.13* (1.01-1.28)	**1.12^+^(1.00-1.28)**	1.04 (0.87-1.21)	

**HDL-Chol (mg/dl)**	55.4(16.2)	0.54* (0.29-0.91)	0.46*(0.26-0.85)	0.78 (0.53-1.08)	

**Systolic BP(mmHg)**	133.5(17.5)	1.58* (1.11-2.24)	1.81**(1.19-2.93)	1.07 (0.80-1.49)	

Diastolic BP **(mmHg)**	75.5(11.7)	0.94 (0.63-1.58)		0.96 (0.63-1.62)	

Insulin user (%)	32.80%	0.96 (0.37-1.93)		1.06 (0.61-1.81)	

**IHD 70 < y.o**.	Mean(SD)	**Hgb A1C = < 7.0 (Gp.OF)**		**7.0 < HgbA1C (Gp.OP)**	

Number of events		25		25	

		Univariate	Multivariate	Univariate	Multivariate

Male/Female	1	1.12 (0.71-1,81)		1.41 (0.83-2.97)	

Age (y.o.)	75.4(4.3)	0.99 (0.88-1.09)		0.83 (0.66-0.99)	

Duration of diabetes (years)	10.18(9.08)	1.02 (0.98-1.06)		1.02 (0.93-1.08)	

Hemoglobin A1C (%)	7.26 (1.15)	1.74 (0.65-5.55)		1.37 (0.77-2.16)	

Triglyceride (mg/dl)	129.2 (62.2)	1.00 (1.00-1.09)		1.01 (0.99-1.04)	

LDL-Chol (mg/dl)	116.1 (30.6)	0.99 (0.78-1.11)		1.05 (0.87-1.24)	

HDL-Chol (mg/dl)	54.7 (16.2)	0.99 (0.73-1.26)		0.79 (0.50-1.18)	

Systolic BP (mmHg)	135.9 (17.1)	0.86 (0.69-1.12)		0.88 (0.61-1.25)	

**Diastolic BP (mmHg)**	72.2 (10.9)	0.65* (0.46-0.95)	0.65*(0.46-0.95)	0.98 (0.59-1.78)	

**Insulin user (%)**	33.90%	0.97 (0.51-1.64)		3.48*(1.42-15.24)	3.48*(1.42-15.24)

**CVA = < 70 y.o**.	Mean(SD)	**Hgb A1C = < 7.0 (Gp.MF)**		**7.0 < HgbA1C (Gp.MP)**	

Number of events		17		15	

		Univariate	Multivariate	Univariate	Multivariate

Male/Female	1.16	1.16 (0.65-2.26)		1.06(0.58-1.98)	

Age (y.o.)	60.9 (7.9)	1.01 (0.94-1.11)		1.02(0.94-1.13)	

**Duration of diabetes (years)**	9.15 (8.22)	0.81*(0.60-0.99)		1.08*(1.01-1.14)	1.06*(1.01-1.11)

HemoglobinA1C(%)	7.38 (1.31)	4.34*(1.07-25.8)	4.11^+^(1.01-13.40)	0.77(0.35-1.35)	

Triglyceride (mg/dl)	146.9(129.5)	1.00 (0.98-1.02)		1.01(0.97-1.07)	

LDL-Chol (mg/dl)	119.2 (34.1)	0.99 (0.96-1.14)		0.98(0.79-1.17)	

**HDL-Chol (mg/dl)**	55.4 (16.2)	0.78 (0.49-1.18)		0.60**(0.35-0.63)	0.43**(0.23-0.78)

Systolic BP (mmHg)	133.5 (17.5)	0.98 (0.71-1.41)		0.99(0.75-1.44)	

Diastolic BP (mmHg)	75.5 (11.7)	1.26 (0.76-2.17)		1.01(0.62-1.83)	

Insulin user (%)	32.80%	0.89 (0.35-1.77)		0.74(0.35-1.18)	

**CVA 70 < y.o**.	Mean(SD)	**Hgb A1C = < 7.0 (Gp.OF)**		**7.0 < HgbA1C (Gp.OP)**	

Number of events		16		22	

		Univariate	Multivariate	Univariate	Multivariate

Male/Female	0.96	1.33 (0.79-2.40)		0.68 (0.36-1.17)	

Age (y.o.)	75.4 (4.3)	1.04 (0.93-1.14)		1.05 (0.93-1.17)	

Duration of diabetes (years)	10.18 (9.08)	1.01 (0.96-1.05)		1.04 (0.98-1.09)	

HemoglobinA1C(%)	7.26 (1.15)	1.27 (0.47-4.29)		0.74 (035-1.32)	

Triglyceride (mg/dl)	129.2 (62.2)	1.01 (0.91-1.07)		1.05 (0.97-1.19)	

LDL-Chol (mg/dl)	116.1 (30.6)	0.96 (0.74-1.10)		1.04 (0.88-1.21)	

**HDL-Chol (mg/dl)**	54.7 (16.2)	0.61*(0.38-0.93)		0.71*(0.47-1.02)	**0.71^+^(0.49-1.02)**

Systolic BP (mmHg)	135.9 (17.1)	0.80 (0.64-1.04)		1.25 (0.91-1.72)	

Diastolic BP (mmHg)	72.2 (10.9)	0.83 (0.55-1.34)		1.09 (0.68-1,85)	

**Insulin user (%)**	33.90%	1.93* (1.05-3.62)	1.93* (1.05-3.62)	1.11 (0.53-2.24)	

^+^P < 0.1, *P < 0.05, **P < 0.01

Clinical characteristics of diabetic individuals and the results of the univariate and multiple regression analyses examining the association between various clinical variables and IHD and CVA risk with stratification by age group. Patients were divided into two age categories: ≤ 70 years of age vs. > 70 years of age. Odds ratios and the corresponding 95% confidence intervals, which are in parentheses following the odds ratios, are shown. Abbreviations: Male/Female: ratio of male patients to female patients; Hemoglobin A1C (%): glycated hemoglobin A1C; LDL-C (mg/dl): Low-density lipoprotein cholesterol; HDL-C (mg/dl): High-density lipoprotein cholesterol; Systolic BP (mmHg): systolic blood pressure; Diastolic BP (mmHg): diastolic blood pressure.

Bold characters indicate that a factor was found to be statistically significant by the univariate or multivariate regression analyses (p < 0.05).

Patients 70 years of age or younger: Univariate analysis revealed that higher systolic blood pressure, higher LDL-C and lower HDL-C were associated with IHD risk among patients in the NF group. However, only systolic blood pressure (OR = 1.81, 95% CI = 1.19-2.93; p = 0.009) and HDL-C level (OR = 0.46, 95% CI = 0.26-0.85; p = 0.006) were confirmed to be significantly associated with IHD risk by the multiple regression analysis.

Patients over 70 years of age: Lower diastolic blood pressure was associated with IHD risk among patients in the OF group (OR = 0.65, 95% CI = 0.48-0.96; p = 0.016). Insulin use was significantly associated with IHD risk in the OP group (OR = 2.27, 95% CI = 1.11-5.89; p = 0.026) (Table 2). The duration of diabetes did not affect IHD risk in either group.

### Multiple regression analysis examining the relationship between clinical variables and the risk of CVA for each group divided by age and glucose control

Table 3 shows the relationship between CVA and each clinical measurement as well as the insulin treatment for each group.

**Table 3:** The stepwise multiple regression analyses to the onset of IHD or CVA

Ischemic Heart Diseases		
= < 70 y.o.	HbA1C = < 7.0 (Gp.MF)	Systolic BP 0.016* HDL-C 0.035* LDL-C 0.049* Gender 0.036*
	7.0 < HbA1C (Gp.MP)	None
70 y.o. <	HbA1C = < 7.0 (Gp.OF)	Diastolic BP 0.033*
	7.0 < HbA1C (Gp. OP)	Insulin 0.006** Age 0.016*
Cerebrovascular Attacks (Stroke)		
= < 70 y.o.	HbA1C = < 7.0 (Gp.MF)	(HbA1C 0.059)
	7.0 < HbA1C (Gp.MP)	HDL-C 0.028*
70 y.o. <	HbA1C = < 7.0 (Gp.OF)	Insulin 0.028* (HDL-C 0.086)
	7.0 < HbA1C (Gp. OP)	None

Patients 70 years of age or younger: CVA tended to occur more frequently among patients with higher HbA1C levels than in those with lower HbA1C levels (OR = 4.11, 95% CI = 1.01-13.4; p = 0.067) in the NF group. Lower HDL-C (OR = 0.43, 95% CI = 0.23-0.78; p = 0.006) and a longer duration of diabetes (OR = 1.06, 95% CI = 1.01-1.11; p = 0.018) were associated with CVA risk among patients in the NP group.

Patients over 70 years of age: Insulin use was associated with CVA risk in the OF group (OR = 2.09, 95% CI = 1.06-4.25; p = 0.028) (Table 2). Univariate analysis revealed a trend toward an association between lower HDL-C levels and CVA in the OF and OP groups, but this association was not statistically significant according to the results of the multiple regression analysis.

### Stepwise regression analysis

Stepwise multiple regression analyses were also performed separately for IHD and CVA risks. The results were very similar to those of the multiple regression analyses described above, except that LDL-C was associated with IHD in the NF group (Table 3).

Regarding IHD risk, higher systolic blood pressure (p = 0.003), lower HDL-C (p = 0.017), and higher LDL-C (p = 0.035) were all associated with IHD risk in the NF group. Insulin use (p = 0.005) and age (p = 0.015) were significantly associated with IHD in the OP group.

Regarding CVA risk, higher levels of hemoglobin A1C were associated with CVA risk in the NF group (p = 0.03), and lower HDL-C (p = 0.004) and the duration of diabetes (p = 0.01) were associated with CVA risk in the NP group. Insulin use (p = 0.038) was associated with CVA risk among patients in the OF group. Lower HDL-C levels tended to be associated with CVA in the OF group (p = 0.056) and the OP group (p = 0.08), although these associations were not statistically significant.

### Influence of insulin therapy on IHD and CVA risk

**Figure 2:**
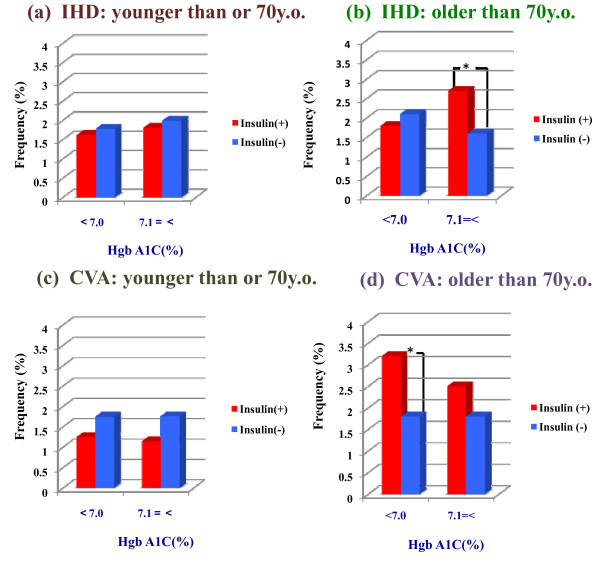
**Effects of age and insulin therapy on the risk of IHD and CVA among diabetic patients stratified by glycemic control (measured by hemoglobin A1C, stratified as ≤ 7.0% vs. > 7.0%)**. Incidence of ischemic heart disease (IHD), left column, and cerebrovascular accident (CVA), right column. Patients with lower hemoglobin A1C values, upper column, and higher hemoglobin A1C values, lower column. (a) Incidence of ischemic heart disease (IHD) (≤ 70 years of age). (b) Incidence of ischemic heart disease (IHD) (> 70 years of age). (c) Incidence of stroke (CVA) (≤ 70 years of age). (d) Incidence of stroke (CVA) (> 70 years of age). These data were adjusted for age and gender. *p < 0.05 Blue column: subjects using insulin. Red column: subjects not using insulin.

The results indicated that insulin therapy was associated with IHD risk in the OP group and with CVA risk in the OF group (Figure 2). Interestingly, patients 70 years of age or younger who used insulin tended to have slightly decreased incidences of IHD and CVA, whereas patients over 70 years of age using insulin tended to have an increased incidence of CVA (Figure 2a and 2b).

Interestingly, insulin use was associated with IHD in the MP group (OR = 4.11, 95% CI = 1.22-8.12; p = 0.018) and with CVA in the FP group (OR = 3.26, 95% CI = 1.12-6.24; p = 0.02).

### Multiple regression analysis examining the relationship between clinical variables and the risk of IHD for each group divided by gender and glucose control

Multiple regression analysis was performed to evaluate the relationship between IHD and each clinical measurement, such as LDL-C level and insulin treatment for each group divided by gender and glucose control levels (HbA1C) (Table 4).

**Table 4:** The relationship on gender between IHD and clinical variables

IHD: Male	Mean(SD)	Hgb A1C = < 7.0 (Gp.MF)		7.0 < HgbA1C (Gp.MP)	
Number of patients		1037		1022	
Number of events		20		26	
		Univariate	Multivariate	Univariate	Multivariate
**Age (y.o.)**	60.9 (7.9)	1.06 (0.97-1.19)		1.10*(1.01-1.22)	1.11*(1.02-1.25)
Duration of diabetes (years)	9.15 (8.22)	1.07(0.98-1.15)		1.09^+^(0.99-1.18)	
HemoglobinA1C(%)	7.38 (1.31)	1.25(0.44-4.60)		1.34(0.72-2.33)	
Triglyceride (mg/dl)	146.9(129.5)	1.00 (0.98-1.01)		1.00(0.98-1.01)	
LDL-Chol (mg/dl)	119.2 (34.1)	1.01 (0.99-1.04)		1.02^+^(1.00-1.04)	
HDL-Chol (mg/dl)	55.4 (16.2)	0.97 (0.92-1.03)		0.99(0.95-1.02)	
**Systolic BP (mmHg)**	133.5 (17.5)	1.06* (1.02-1.11)	1.06* (1.02-1.11)	0.99(0.94-1.04)	
Diastolic BP (mmHg)	75.5 (11.7)	0.95 (0.89-1.02)		1.03(0.95-1.12)	
**Insulin user (%)**	32.80%	1.74 (0.55-2.77)		4.01**(1.15-7.38)	4.11**(1.19-8.12)
**IHD:Female**	Mean(SD)	Hgb A1C = < 7.0 (Gp.OF)		7.0 < HgbA1C (Gp.OP)	
Number of patients		816		1140	
Number of events		19		17	
		Univariate	Multivariate	Univariate	Multivariate
Age (y.o.)	75.4 (4.3)	1.00 (0.96-1.06)		0.99 (0.95-1.05)	
**Duration of diabetes (years)**	10.18 (9.08)	0.96*(0.93-0.98)	0.96*(0.93-0.98)	0.92*(0.84-0.99)	0.92*(0.84-0.99)
HemoglobinA1C(%)	7.26 (1.15)	1.01 (0.78-1.31)		0.84 (0.61-1.10)	
Triglyceride (mg/dl)	129.2 (62.2)	1.01 (0.95-1.07)		0.99 (0.96-1.02)	
LDL-Chol (mg/dl)	116.1 (30.6)	1.00 (0.98-1.03)		1.00 (0.95-1.05)	
HDL-Chol (mg/dl)	54.7 (16.2)	0.97(0.92-1.02)		0.92(0.80-1.03)	
Systolic BP (mmHg)	135.9 (17.1)	1.04 (0.92-1.16)		1.05 (0.91-1.18)	
Diastolic BP (mmHg)	72.2 (10.9)	0.93 (0.85-1.03)		1.05 (0.94-1.15)	
Insulin user (%)	33.90%	1.08^+^(1.01-1.15)		6.27^+^(0.91-14.24)	
**CVA: Male**	Mean(SD)	Hgb A1C = < 7.0 (Gp.MF)		7.0 < HgbA1C (Gp.MP)	
Number of events		22		16	
		Univariate	Multivariate	Univariate	Multivariate
Age (y.o.)	60.9 (7.9)	0.99 (0.92-1.08)		1.05(0.92-1.25)	
Duration of diabetes (years)	9.15 (8.22)	1.03^+^(0.99-1.06)		1.01(0.99-1.02)	
HemoglobinA1C(%)	7.38 (1.31)	1.68(0.74-4.33)		0.92(0.72-1.24)	
Triglyceride (mg/dl)	146.9(129.5)	1.00 (0.98-1.01)		0.99(0.97-1.01)	
LDL-Chol (mg/dl)	119.2 (34.1)	1.01 (0.99-1.04)		1.02(0.98-1.05)	
HDL-Chol (mg/dl)	55.4 (16.2)	0.96^+^(0.91-1.01)		0.99(0.92-1.05)	
Systolic BP (mmHg)	133.5 (17.5)	1.01 (0.95-1.06)		0.99(0.92-1.08)	
Diastolic BP (mmHg)	75.5 (11.7)	0.96 (0.90-1.05)		1.05(0.93-1.22)	
Insulin user (%)	32.80%	4.44 (0.35-6.77)		1.01(0.35-1.18)	

**CVA:Female**	Mean(SD)	Hgb A1C = < 7.0 (Gp.OF)		7.0 < HgbA1C (Gp.OP)	

Number of events		11		21	
		Univariate	Multivariate	Univariate	Multivariate
Age (y.o.)	75.4 (4.3)	1.00 (0.93-1.12)		1.35*(1.00-1.85)	
**Duration of diabetes (years)**	10.18 (9.08)	0.97*(0.95-0.99)	0.97*(0.95-0.99)	0.94*(0.88-0.98)	0.95*(0.89-0.99)
HemoglobinA1C(%)	7.26 (1.15)	7.48 (0.70-22.8)		0.94 (0.65-1.30)	
Triglyceride (mg/dl)	129.2 (62.2)	1.01 (0.91-1.07)		1.01 (0.97-1.03)	
LDL-Chol (mg/dl)	116.1 (30.6)	0.96 (0.74-1.10)		1.02 (0.99-1.05)	
HDL-Chol (mg/dl)	54.7 (16.2)	0.99(0.92-1.05)		0.99(0.94-1.03)	
Systolic BP (mmHg)	135.9 (17.1)	1.02 (0.90-1.10)		0.88 (0.76-0.97)	
Diastolic BP (mmHg)	72.2 (10.9)	1.03 (0.94-1.17)		1.02 (0.91-1.16)	
**Insulin user (%)**	33.90%	0.93 (0.75-1.12)		3.26*(1.12-6.24)	3.29*(1.13-6.42)

+P < 0.1, *P < 0.05, **P < 0.01

Clinical characteristics of diabetic individuals and the results of the univariate and multiple regression analyses examining the association between various clinical variables and IHD and CVA risk with stratification by gender group. Odds ratios and the corresponding 95% confidence intervals, which are in parentheses following the odds ratios, are shown.

Male patients: IHD was associated with high systolic blood pressure in the male/fair glycemic control group, age in the male/poor control group. Interestingly, insulin use was associated with IHD in the male/poor group (OR = 4.11, 95% CI = 1.22-8.12; p = 0.018).

Female patients: IHD was associated with a short duration of diabetic history in the female/fair and female/poor groups.

### Multiple regression analysis examining the relationship between clinical variables and the risk of CVA for each group divided by gender and glucose control

Multiple regression analysis was performed to evaluate the relationship between CVA and each clinical measurement and insulin treatment for each group divided by gender and glucose control levels (HbA1C) (Table 4).

Male patients: CVA was not significantly associated with any variables.

Female patients: CVA was associated with a short duration of diabetic history in the female/fair and female/poor groups. Insulin use was associated with CVA in the female/poor group (OR = 3.26, 95% CI = 1.12-6.24; p = 0.02).

## Discussion

In the present study, IHD was associated with a higher systolic blood pressure and a lower HDL-C in patients in the 70 years of age or younger patients with fair glycemic control and a lower diastolic blood pressure in older patients with fair glycemic control. Insulin use was associated with IHD in the **OP **group, whereas it was associated with CVA in the **OF **group. CVA was associated with lower HDL-C and a longer duration of diabetes in patients in the NP group. The results obtained by stepwise analysis were similar, except that LDL-C was associated with IHD in patients in the NP group. In the elderly, insulin use and glycemic control may contribute differently to IHD and CVA risks.

The frequency of diabetes increases with age, and there are many elderly diabetic individuals. However, the risk factors for IHD and CVA have not been clearly defined in elderly diabetics. Furthermore, there is insufficient clinical evidence regarding the effects of insulin therapy on IHD and CVA risks in elderly patients [13,14]. Therefore, this study examined the effect of insulin therapy on IHD and CVA risks among elderly diabetics. This study also examined the possibility that IHD and CVA risk factors varied by age.

In the present study, differences in the IHD and CVA risks by gender were not evident. IHD was associated with high systolic blood pressure in the MF group, age in the MP control, short duration of diabetic history in the FF and FP groups. Insulin use was associated with IHD in the MP group and with CVA in the FP group (OR = 3.26, 95% CI = 1.12-6.24; p = 0.02). CVA was associated with short duration of diabetes in both female groups.

### IHD risk factors

American guidelines for diabetic control suggest that diabetic individuals under 70 years of age have a risk of developing IHD similar to that for non-diabetic individuals having a prior myocardial infarction [8], and the results from the present study support this concept (Figure 2). We found that the incidence of IHD among diabetic Japanese was relatively high and comparable with that of IHD in Western countries, such as the U.K. [3,15]. The incidence of IHD in our study was approximately three times higher than the incidence rates previously reported in Japanese trials, such as Mega study investigating patients under 70 years of age, not all of whom had diabetes [16,17].

In the present study, among diabetic individuals in the NF group, higher systolic blood pressure and lower HDL-C were associated with a risk of IHD. Systolic blood pressure, not diastolic pressure, was confirmed as a classical IHD risk factor from previous studies [18-20]. The age of diabetic patients in those reports was less than 70. Stepwise regression analysis confirmed the association of high LDL-C and low HDL-C with a risk of IHD in the NF group; however, the significance of the LDL-C association was not confirmed by multiple regression analysis [21]. The use of anti-dyslipidemia agents, such as statins, with their pleiotropic effect may affect this difference [21]. Insulin use tended to decrease the incidence of IHD in patients in the NF group (Table 2).

Among older diabetic individuals with fair glycemic control (the OF group), lower diastolic blood pressure was associated with IHD. Insulin use was associated with IHD in the OP group. Coronary circulation depends on diastolic blood flow, and the isolated systolic hypertension (with lower diastolic blood pressure) values may reflect aortic atherosclerosis, which is common among the elderly.

Interestingly, insulin therapy was associated with IHD in the OP group. The duration of diabetes was longer among insulin users than among non-users in the NF and NP groups; however, there was no difference in diabetes duration between the OF compared to the OP group. Overall, the combination of higher plasma glucose and insulin use may progress atherosclerosis and subsequently increase the risk of IHD among elderly diabetic individuals. However, maintaining good glycemic control via insulin use could help prevent IHD. Because patients with a history of IHD and/or CVA were excluded from the study, few patients in our cohort used antiplatelet agents (< 10% of patients).

Conversely, IHD was associated with high systolic blood pressure in the MF group, age in the MP control, and short duration of diabetic history in the FF and FP groups. Insulin use was associated with IHD in the male/poor group. Females usually develop complications of atherosclerotic diseases, such as IHD or CVA, 10-15 years later than males [22], which may partially be due to the prevention of atherosclerosis progression by estrogen. However, previous reports have indicated that gender does not affect the age of onset of atherosclerotic disease in individuals with diabetes [23,24]. Our observation that a short duration of diabetic history is associated with IHD and CVA in females may reflect these phenomena. A detailed mechanism was not obvious in the present study and should be evaluated in future research. Regarding gender differences and laboratory findings in Japanese populations, it was recently suggested that hsCRP levels increase continuously across the fasting plasma glucose (FPG) spectrum, starting from the lowest FPG in both men and women, but the increase in hsCRP levels is greater in women than men. Moreover, higher CRP gamma-glutamyl transferase (GGT) levels are synergistically associated with the metabolic syndrome and insulin resistance, independently of other confounding factors in the general population [25,26].

### CVA risk factors

The incidence of CVA is higher in East Asian individuals, such as the Japanese, than in Caucasians. Thus, the incidence of CVA was higher in the present study than those reported in previous Western studies, but it was comparable to that observed in prior Japanese studies, such as JDCS [27]. The differences in eating habits, diabetic complications, and older average age observed in the present study may have led to these differences.

Among patients in the NP group, low HDL-C was associated with CVA. This result is consistent with previous reports; however, the relationship between the HDL-C level and CVA has been described recently [9,28].

Among patients in the OF group, insulin use was associated with CVA (OR = 2.09). Although the underlying mechanism is still unknown and the higher frequency of CVA in Japan may affect the data, our preliminary findings show that hypoglycemia occurred more frequently among individuals in the OF group than those in the OP group. Hypoglycemia increases stroke risk [5,29]. In the strict glucose control group of the ACCORD study, morbidity rates increased in the form of severe hypoglycemia and weight gain.

In the OP group, glycemic control was worse among insulin users than among non-users. The duration of diabetes did not differ between the OF and OP groups. There has been a drastic increase in the elderly population in the last several decades. Therefore, understanding the characteristics of lifestyle-related diseases in the elderly is important to maintain their good health. Postprandial hyperglycemia is common in the elderly, and hyperosmolar nonketotic hyperglycemia often complicates the course of diabetes in the elderly. Insulin therapy reduces glucose toxicity and is necessary in some elderly diabetic patients. Strict blood glucose control including insulin therapy is necessary to prevent the progression of diabetic microangiopathies. However, insulin induces smooth muscle cell proliferation and may lead to the progression of atherosclerosis [30]. Muis et al. focused on type 1 diabetes patients (instead of type 2) to minimize the effects of insulin resistance [31]. They found that the cumulative dose of regular insulin was significantly related to carotid intima-media thickness. They observed a similar relationship between the use of intermediate-acting insulin with carotid intima-media thickness and concluded that the cumulative dose of insulin was a risk factor for atherosclerosis. Insulin contributes to cellular senescence and causes aging in organisms, such as mice [32,33]. The detrimental effects of insulin therapy, such as hypoglycemia leading to stroke, was more evident in the elderly in the present study. Unfortunately, a detailed analysis was not possible because of the fact that patients' insulin regimens (dose and type of insulin) change frequently. Future studies should examine the effects of insulin. CVA was associated with short duration of diabetes in the FF and FP groups, and insulin use was associated in the FP group. The results that short duration of diabetic history is associated with CVA in the female may be explained similarly as to IHD, as drastic effect of menopause [34].

### Limitations

Treatment for diabetes was based on data recorded at the time of enrollment. Patients were followed for 2 years, and we could not analyze the detailed mechanisms underlying insulin therapy on the risk of IHD and CVA.

## Conclusions

The present study suggests that the risk factors for IHD and CVA in diabetic individuals change with age and gender and perhaps with a patient's degree of glycemic control. Insulin use has a potential role in preventing IHD but may also be a risk factor for CVA among the diabetic elderly. Therefore, although the treatment of diabetes is obviously important, insulin therapy for glycemic control should be carefully considered in those elderly patients. Treatment modalities that reduce the adverse effects of insulin without sacrificing its glycemic controlling effects would be of particular interest in the treatment of elderly diabetic individuals.

## Abbreviations list

IHD: ischemic heart disease; CVA: cerebrovascular accident; T2DM: type2 diabetes mellitus; UKPDS: the United Kingdom Prospective Diabetes Study; ACCORD: Action to Control Cardiovascular Risk in Diabetes; ADVANCE: Action in Diabetes and Vascular Disease: Preterax and Diamicron Modified Release Controlled Evaluation; JCDM: Japan Cholesterol and Diabetes Mellitus Investigation; NF: under 70 years of age with fair glycemic control; NP: under 70 years of age with poor glycemic control; OF: over 70 years of age with fair control; OP: over 70 years of age with poor control; MF: males with fair control; MP: males with poor control; FF: females with fair control; FP: females with poor control.

## Competing interests

The authors declare that they have no competing interests.

## Authors' contributions

TH, SK, HI, HW, TO, HS, YK, YH, MY and KI participated in the design of the study and carried out the cohort study in their hospitals and related hospitals. HN and KK participated in the design of the study and performed the statistical analysis. TH also conceived of the study, and participated in its coordination. All authors read and approved the final manuscript.
